# Engineering Pectin Biobased Films with Bacterial Cellulose Nanostructures for Enhanced Food Packaging Performance

**DOI:** 10.1021/acsfoodscitech.5c00405

**Published:** 2025-08-20

**Authors:** Mirva Sarafidou, Erminta Tsouko, Anastasios Giannoulis, Demetres Briassoulis, George E. Baltatzis, Ioannis P. Trougakos, Theofania Tsironi, Apostolis Koutinas

**Affiliations:** † Department of Food Science and Human Nutrition, 68995 Agricultural University of Athens, Iera Odos 75, Athens 11855, Greece; ‡ Division of Genetics & Biotechnology, Department of Biology, National and Kapodistrian University of Athens, 15784 Athens, Greece; § Laboratory of Farm Structures, Department of Natural Resources Management and Agricultural Engineering, Agricultural University of Athens, Iera Odos 75, 11855 Athens, Greece; ∥ Imaging Core Facility, National and Kapodistrian University of Athens, 11584, Athens Greece; ⊥ Division of Cell Biology and Biophysics, Department of Biology, National and Kapodistrian University of Athens, Athens 15784, Greece

**Keywords:** sugar beet pulp, biorefinery context, bacterial cellulose nanostructures, biobased pectin films, food packaging materials

## Abstract

This study developed pectin-based (Pec) films reinforced with microfibrillated cellulose (MFC) and bacterial cellulose nanostructures (BNC) produced via acid (BNC-A) or enzymatic (BNC-E) processing for sustainable food packaging. Sugar beet pulp served as a renewable resource for bacterial cellulose production (3.9 g/L) and food-grade pectin (galacturonic acid = 76.9%). Transparency and optical properties of films were influenced by BNCs incorporation (*p < 0.05*). BNC-reinforced films blocked more than 95% of the UVA/UVB radiation. The contact angle ranged within 74.6–106.7°, with BNC-A-reinforced films demonstrating the highest hydrophobicity. Water vapor permeability ranged within 1.78 × 10^–7^-2.07 × 10^–7^ g/m·h·Pa, with insignificant differences between the cellulose-reinforced and Pec films (*p > 0.05*). BNC-A incorporation improved the film’s mechanical profile, with tensile strength, elongation at break, and Young’s modulus rising by 39.7, 53.6, and 54.0%, respectively, over Pec films. Overall, Pec films reinforced with BNCs emerge as strong candidates for sustainable food packaging, combining mechanical strength, efficient UV-protection, and tunable water interaction, supporting eco-friendly packaging alternatives.

## Graphical abstract

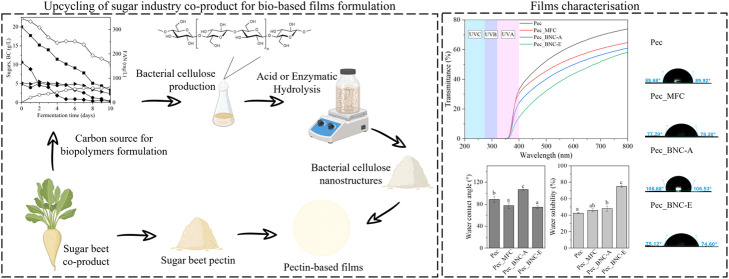

## Introduction

Sugar beet (*Beta* *vulgaris* subsp. *vulgaris*) production capacity reached 110.3 million tonnes in 2023 (European Union), accounting for over 39% of global sugar beet production.^1^ Sugar beet pulp (SBP), the primary byproduct of sugar beet processing, is generated during the extraction of sugar-rich juice through diffusion and pulp pressing, followed by additional treatment for further sugar recovery.^2^ SBP predominantly consists of structural carbohydrates, including cellulose, hemicellulose, and pectin, which collectively represent >50% of the dry biomass. Residual sugars, derived from remaining sucrose, account for 4–11% of the composition.^2,3^ Currently, SBP is primarily valorized through low-value processes, such as animal feed (typically via pelletization), bioethanol production, and energy generation.^4,5^

Pectin is a structural polysaccharide, primarily composed of galacturonic acid (GalA) units (>65%), linked by α-1,4-glucosidic bonds. It is commonly extracted from various agro-industrial residues, including citrus peels, apple pomace, and SBP.^6–8^ Among the various pectin types, SBP-derived pectin is classified as high methoxy pectin (degree of esterification, DE > 50%), with enhanced emulsification properties, making it suitable for use as a thickening, emulsifying, and encapsulating agent in food-related applications.^9^

Pectin has found extensive application as a biobased material for food packaging, particularly in the form of films and coatings.^10–13^ Despite their promising potential, pectin-based films often exhibit limitations, particularly in terms of mechanical properties, such as low tensile strength and limited elasticity, which can hinder their practical applicability in food packaging systems.^14,15^ Additionally, while pectin films can offer antioxidant properties, notably through compounds like ferulic acid, they may lack sufficient barrier properties and durability, which are critical for effective food preservation.^16,17^ The incorporation of bacterial cellulose (BC) into pectin-based films has emerged as an effective strategy to overcome the limitations of pure pectin films related to mechanical profile and barrier properties.^18–20^ BC is a natural biopolymer that is produced efficiently by acetic acid bacteria. While BC shares the same chemical structure as plant-based cellulose, it exhibits superior mechanical properties, higher crystallinity, and enhanced water-holding capacity. BC is biodegradable, biocompatible, and possesses a unique network structure that contributes to its exceptional properties.^21–23^ Structural modification of BC could offer further advantages for biobased films reinforcement. The hydrolysis/modification process (with acids or enzymes) breaks down the cellulose fibers into smaller fragments, resulting in nanocrystals or nanofibrils, which present enhanced surface area and reactivity.^21,24^ Additionally, the smaller dimensions allow for better integration within the film matrix, improving the overall homogeneity and functionality.^20^

Conventional food packaging materials predominantly derive from fossil fuels and are widely utilized due to low cost, high durability, mechanical strength, transparency, and low toxicity.^25^ However, plastic packaging possesses significant environmental concerns, prompting extensive research on sustainable and eco-friendly alternatives. Natural biopolymers, such as polysaccharides derived from agro-industrial waste streams or produced through microbial fermentation, have the potential to meet the increasing demand for sustainable packaging solutions by providing biodegradability, renewability, and functional versatility.^26–28^

The incorporation of cellulose into pectin-based matrices was previously examined. Recent work by Viana et al.^19^ demonstrated that TEMPO-oxidized nanocellulose from SBP significantly improved the mechanical and barrier properties of pectin-rich films. To the best of our knowledge, the incorporation of BNCs into pectin-based films derived from SBP has not been investigated. This study explores the sustainable utilization of SBP for the dual purpose of pectin recovery and BC synthesis, targeting the development of biobased films for food packaging. Two distinct BC forms were generated via chemical and enzymatic routes and subsequently embedded in the pectin matrix. The resulting composite films were evaluated for their optical, chemical, and mechanical attributes. The proposed biorefinery approach showcases an effective SBP valorization pathway: free sugars are converted to BC, while the pectin-rich residue is repurposed for high-value pectin extraction. This integrated approach promotes waste reduction and supports the production of functional biopolymers applicable in the food, pharmaceutical, and biotechnology sectors.

## Materials and Methods

### Raw Material

SBP was kindly provided by Dimitriaki S.A. (Thessaloniki, Greece), ground, and stored at room temperature. The chemical composition of SBP (w/w) was as follows: glucan (27.9%), hemicellulose (25%), pectin (19.1%), free sugars (10.9%), protein (9.1%), ash (3.7%), lignin (2.3%), and lipid (0.9%). Free sugars are composed of 82.6% sucrose, 13.8% glucose, and 3.7% fructose.^2^

### Free Sugars Preparation Using SBP

An aqueous suspension of SBP (1:20 w/v) was heated to 40 °C for 2 h on a hot plate stirrer (Witeg Labortechnik GmbH, Germany) under continuous agitation. Next, the suspension was centrifuged at 9000 rpm for 15 min to separate the sugar-rich liquid fraction from the pectin-rich solid residue (SBPR). Both the liquid phase and the SBPR were collected for further use.

### Pectin Extraction and Characterization

Pectin was extracted following the procedure described by Tsokri et al.^11^ with slight modifications. An aqueous suspension of SBPR (1:20 w/v) was prepared, and nitric acid (5 M) was added until the pH reached 2.0. Hot acid-assisted extraction was performed at 90 °C for 2 h under continuous stirring (300 rpm, Witeg MSH-D, Germany). The liquid phase was separated by filtration (Whatman filter paper No. 1), followed by pH adjustment to 3.5 using 5 M NaOH and addition of 2:1 (v/v) ethanol. After overnight precipitation at 4 °C, pectin was recovered by filtration, washed with ethanol, and freeze-dried. Pectin yield was calculated as the percentage of dry pectin-rich extract (g) divided by the initial amount of SBPR (g). The uronic acid content was determined using the m-hydroxybiphenyl colorimetric method, with GalA as the standard, as described by Melton and Smith.^29^ The DE was determined by titration, as described by Tsokri et al.^11^

### Fermentation for BC Production

*Komagataeibacter sucrofermentans* DSM 15973 (Leinbniz-DSMZ, Germany) was used for BC production. The media preparation, BC fermentation (in 250 mL Erlenmeyer flasks), and BC purification were based on Sarafidou et al.^21^ with slight changes. The sugar-rich stream of SBP, 3-fold concentrated (rotary evaporator, Büchi Labortechnik AG, Switzerland) to reach 2% carbon concentration, served as a carbon source in the fermentation media. This stream was filter-sterilized (Polycap AS, Whatman Ltd., 0.22 μm pore size), and supplemented with a separate media (sterilized at 121 °C for 20 min) containing nitrogen sources (5 g/L yeast extract and 5 g/L bacteriological peptone) and a mineral solution (2.7 g/L Na_2_HPO_4_ and 1.15 g/L citric acid). Subsequently, the fermentation media was inoculated with 10% (w/w) of a preculture medium (identical in composition with the fermentation media but using glucose as the carbon source, incubated at 200 rpm for 24 h) and the flasks were incubated at 30 °C for 10 days. BC was harvested from the culture broth, treated with 1 M NaOH (24 h under continuous agitation at 180 rpm) to remove impurities, and washed repeatedly with tap water until a neutral pH was monitored. BC was then freeze-dried (New Life Scientific, US), weighed, ground to 1 mm using a hammer mill (ECB mill, London), and stored at room temperature for future use.

Sampling was conducted daily to monitor the consumption of free amino nitrogen and sugars, as well as the production of BC. Free amino nitrogen was quantified using the ninhydrin method.^30^ Sugar concentrations (sucrose, glucose, and fructose) were determined by using a high-performance liquid chromatography (HPLC) system (Shimadzu, Japan) equipped with a refractive index (RI) detector. The analysis was performed on a Phenomenex Rezex ROA-organic acid H^+^ column (300 mm length × 7.8 mm diameter) at 65 °C, with a 10 mM H_2_SO_4_ solution as the mobile phase and a flow rate of 0.6 mL/min.

### Preparation of Bacterial Cellulose Nanostructures (BNCs)

#### Acid Hydrolysis

BC acid hydrolysis was performed following the procedure described by Sarafidou et al.^21^ Briefly, 4 g of BC were suspended in distilled water and homogenized (IKA Ultra-Turrax T25 basic at 15,500 rpm for 4 min). H_2_SO_4_ (95–97%, Honeywell) was added to achieve a final concentration of 50% (w/w). The hydrolysis was carried out at 55 °C for 48 h under continuous agitation (500 rpm), with termination achieved by adding five volumes of distilled water. Bleaching was performed with 30% H_2_O_2_ at a specified amount for 1 h at 200 rpm, followed by three rounds of centrifugation (7500 rpm, 15 min, 4 °C). The BC slurry was then suspended in distilled water, subjected to ultrasonication at 60 kHz and 300 W (Sonoplus 3200, Germany) for 6 min, and neutralized using dialysis bags (Medicell Membranes Ltd., cutoff 12–14 kDa). The final suspensions were lyophilized (New Life Scientific), ground to produce a homogeneous material (BNC-A), and stored for further use.

#### Enzymatic Modification of BC

The enzymatic modification of BC was performed as follows. Water suspensions of BC (20 g/L) were homogenized (IKA Ultra-Turrax T25 basic at 10000 rpm for 4 min) and sterilized. Enzymatic reactions were conducted on a hot plate stirrer (Witeg Labortechnik GmbH, Germany) (55 °C, 200 rpm) using an initial enzymatic activity of 50 U/g BC (*Trichoderma reesei* cellulase, ≥ 700 U/g, Sigma-Aldrich) at pH 5.0 for 24 h. The mixtures were then centrifuged (9000 rpm, at 4 °C for 20 min), and the treated material was collected, washed, and centrifuged iteratively to remove any residual compounds. BNC was lyophilized (New Life Scientific, US), ground to produce a homogenized material, and stored until further use (BNC-E).

#### Structural Characterization of Cellulosic Structures

Scanning electron microscopy (SEM) was performed to characterize the structural properties of the samples. BC, MFC, and BNCs were coated with a thin layer of gold using a sputter coater (EM ACE200, Leica Microsystems) and analyzed using a Hitachi SEM (SU3800) operating at an accelerating voltage of 5 kV at a magnification ranging from 5000× to 20,000×.

### Films Formulation

Pectin-based films (Pec) were prepared by gradually dissolving 3 g of pectin in 100 mL of distilled water at 70 °C under continuous stirring until complete solubilization. The solution was then cooled, and 30% (w/w) glycerol and 5% (w/w_pectin_) cellulose (based on pectin weight) were added. Three types of cellulose were used: commercial microfibrillated cellulose (MFC, Borregaard AS, Norway) to produce Pec_MFC films, BNC-A to produce Pec_BNC-A films, and BNC-E to produce Pec_BNC-E films. The mixtures were stirred at ambient temperature for 30 min to ensure homogeneity. A 25 mL aliquot of each mixture was then cast into Petri dishes (90 × 15 mm^2^) to form films. The films were dried in an oven at 50 °C for 24 h, peeled off, and stored in a desiccator.

### Film Characterization

#### Attenuated Total Reflectance-Fourier Transform Infrared Spectroscopy (ATR-FTIR)

The functional groups present in extracted pectin, MFC, BNC-A, BNC-E, and formulated films were determined by the ATR-FTIR technique performed on a Bruker Optik Fourier instrument (Tensor II, Equinox 55S) equipped with an ATR diamond accessory from SENS-IR and a press. A total of 50 scans were performed at ambient temperature in a wavenumber range of 4500–750 cm^–1^ (4 cm^–1^ resolution).

The degree of methyl-esterification (DM) of extracted pectin was estimated using the peak areas from COOCH_3_ (∼1600 cm^–1^, *I*_A_) and COOH (∼1720 cm^–1^, *I*_B_) groups (DM), according to the eq 1.^31^

1 $$\mathrm{DM}=\frac{I_{A}}{I_{A}+I_{B}}\times 100$$

#### Color

The color of the films was expressed as lightness (L*), redness (a*), and yellowness (b*) parameters through the CIELAB color system. The color parameters were measured from 20 different points of the films using a colorimeter (Eye-one Pro, X-Rite, Grand Rapids, MI) with a light source D65 and observation angle 10°. A white standard (L* = 98.96, a*= −0.18 and b* = −0.12) was used as a background. The total color difference, whiteness index, and yellowness index were determined using eqs 2, 3, and 4.^32^

2 $$\mathrm{total}\;\mathrm{color}\;\mathrm{difference}=\sqrt{{\left(L-L_{0}\right)}^{2}{+\left(a-a_{0}\right)}^{2}+{\left(b-b_{0}\right)}^{2}}$$

3 $$\mathrm{whiteness}\;\mathrm{index}=100-\sqrt{{\left(100-L\right)}^{2}+a^{2}+b^{2}}$$

4 $$\mathrm{yellowness}\;\mathrm{index}=\frac{142.86\times b^{2}}{L^{*}}$$

where *L*_0_, *a*_0_, and *b*_0_ are the *L*, *a*, and *b* values of neat pectin film.

#### UV–vis Transmittance of the Pectin-Based Films

The films were cut into pieces (1 × 4 cm^2^), and the average thickness (*L*, mm) was recorded by three measurements at different points, using a calibrated digital micrometer (Mitutoyo). Each film was placed in a ultraviolet–visible (UV–vis) spectrophotometer (Shimadzu UV–1900i) and the spectra were recorded (200–800 nm wavelength) from five different replicates, using air as a reference. Transparency (*T*%)^33^ and UV-blocking %^34^ were calculated according to the following eqs 5, 6, 7.

5 $$T\%=\log\left(\%T_{600\mathrm{nm}}\right)/L$$

6 $${\mathrm{UV}}_{A}-\mathrm{blocking}\%=100-T_{{\mathrm{UV}}_{A}}$$

7 $${\mathrm{UV}}_{B}-\mathrm{blocking}\%=100-T_{{\mathrm{UV}}_{B}}$$

where *T*_600 nm_ is the transmittance of each film determined at 600 nm, *T*_UVA_ and *T*_UVB_ are the average transmittance in the UV-A (315–400 nm) and UV–B (280–315 nm) spectra, respectively.

#### Water Content and Solubility

The water content and solubility of films were assessed using the method outlined by Athanasopoulou et al.^35^ with slight modifications. The films were cut into pieces (2 × 3 cm^2^), weighed (*W*_0_), dried at 80 °C for 24 h, cooled down into a designator, and reweighed (*W*_d_). The dried films were then immersed in distilled water (50 mL) and after 24 h, the swollen samples were dried for 24 h at 80 °C to determine the final weight (*W*_ds_). All the measurements were performed in triplicates. The water content and solubility were calculated as follows.

8 $$\mathrm{water}\;\mathrm{content}=\frac{W_{0}-W_{d}}{W_{d}}\times 100\%$$

9 $$\mathrm{solubility}=\frac{W_{d}-W_{\mathrm{ds}}}{W_{d}}\times 100\%$$

#### Contact Angle

The contact angle of each film was recorded based on an ASTM D5946 method^36^ using a Theta Flow Optical Tensiometer (Biolin Scientific, Gothenburg, Sweden) and sessile drop. A drop of 4 μL of distilled water was placed on the film’s surface at 25 °C. Contact angle was measured at six different points of the film’s surface, and the average was recorded.

#### Water Barriers

The water barriers of the formulated films were identified using the method reported by De Rezende et al.^37^ with some modifications. Cut films with known thickness (*T*_h_, m) were used to seal plastic cups (3 cm inner diameter and 3.5 cm depth) filled with 5 mL of deionized water.^38^ The samples were placed into a designator, followed by mass loss recording (*G*, g) every 2 h (*t*, h) over 8 h, followed by an additional measurement at 24 h. The difference between the partial pressure of water on both sides of the film (Δ*P*) was equal to 3.179 kPa, as the relative humidity values of the inner and outer surfaces of the film are 100% (3.179 kPa vapor pressure) and 0% (0 kPa vapor pressure), respectively. The water vapor transmission rate (WVTR) and water vapor permeability (WVP) were calculated according to the eqs 10 and 11, respectively.

10 $$\mathrm{WVTR}=\frac{G}{t\times A}$$

11 $$\mathrm{WVP}=\frac{\mathrm{WVTR}\times T_{h}}{\Delta P}$$

where *A* is the area of the plastic cup’s mouth.

#### Mechanical Properties

Each film sample was evaluated for its mechanical properties according to the ISO 527–3:2018 with slight modifications, using a universal testing machine (Instron, Model 5900). Films were cut into strips (1 × 6 cm^2^) with a thickness of 80 ± 10 μm. The measurements were carried out at 10 mm/min separation speed, 23 °C, and 50% relative humidity. Tensile strength (MPa), elongation at break (%), and Young’s modulus (MPa) were calculated automatically by the Instron Bluehill 3′ Software. Five samples of each film were measured, and the average values along with the standard deviations were recorded.

### Statistical Analysis

Statistical analysis between the values was conducted using STATGRAPHICS software (STATGRAPHICS Centurion 293 XVII, specifically Version 17.2.00). A one-way analysis of variance and Duncan’s multiple range test with a 95% confidence interval were performed. The results were expressed as the mean ± standard deviation of three replicates.

## Results and Discussion

### BC Production and Pectin Recovery Valorizing Renewable SBP

The sugar-rich liquid fraction from SBP was used as a carbon source for BC production under batch fermentation (Figure 1). Sucrose was entirely consumed within 5 days of fermentation, accompanied by the assimilation of 93.2 mg/L of free amino nitrogen, leading to a BC concentration of 3.3 g/L, with a yield of 0.2 g BC/g total sugars and a productivity of 0.7 g/L/day. Notably, the increased levels of glucose and fructose observed on day 2 likely originated from the enzymatic or spontaneous hydrolysis of sucrose into its monosaccharide components. After that point, *Komagataeibacter sucrofermentans* primarily metabolized glucose, while fructose consumption proceeded at a slower rate. After 10 days of fermentation, more than 90% of glucose and 55% of fructose were consumed, with BC production being slightly increased (3.9 g/L). These findings align with Adamopoulou et al.,^39^ who reported that BC production is most efficient when sucrose serves as the main carbon source, followed by glucose and fructose. Tsokri et al.^11^ employed SBP hydrolysate (composed mainly of glucose) in fed-batch fermentations, achieving a maximum BC production of 3.0 g/L, while Sarafidou et al.^21^ employed batch fermentations with SBP hydrolysate, attaining a 4.6 g/L BC, with a yield of 0.33 g/g and a productivity of 0.65 g/L/day, highlighting the influence of fermentation mode and process conditions on BC synthesis efficiency. Overall, these results underscore the potential of SBP-derived sugars as renewable carbon sources for sustainable BC production, demonstrating effective sucrose utilization and subsequent glucose-driven biosynthesis.

**Figure 1:**
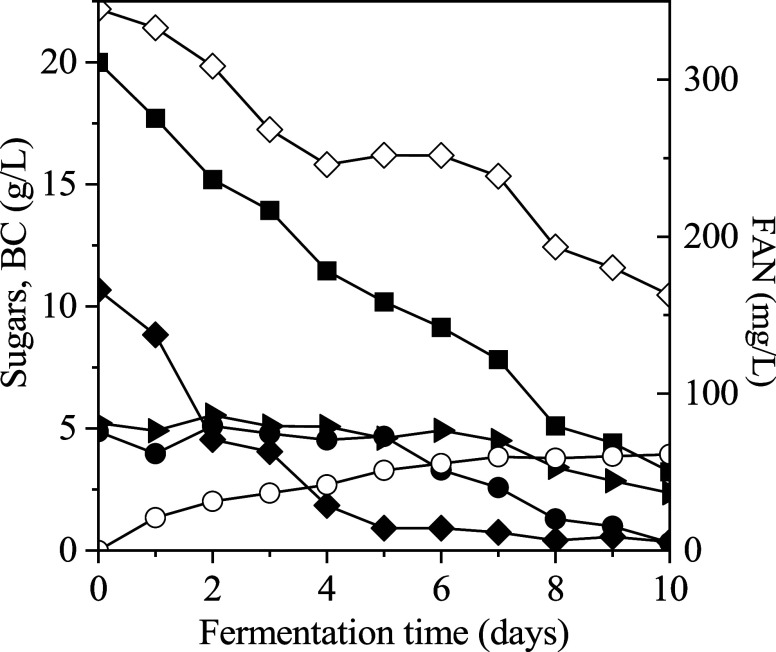
Valorization of free sugars extracted from SBP, as a carbon source for BC production by the bacterial strain *K. sucrofermentans*. Total sugars (■); sucrose (⧫); glucose (●); fructose (▶); BC (○); free amino nitrogen (FAN) (◊).

The produced BC was further treated, leading to the production of BNC-A and BNC-E, followed by SEM characterization (Figure 2). The SEM micrographs of BC, hydrolyzed BC structures, and MFC are shown in Figure 2. The SEM micrograph of native BC exhibited a dense, multilayered network of ribbon-like nanofibers (Figure 2A). Fibrils appear continuous and well-entangled with diameters in the range of 51–98 nm, which is typical of BC. BNC-A subjected to H_2_SO_4_ hydrolysis (Figure 2C) presented clear evidence of network disruption. The fibrils appeared thinner (45–80 nm) and were highly fragmented. Aggregated clusters and compacted regions were evident, likely resulting from the freeze-drying process, which can induce the capillary-driven collapse and aggregation of nanoscale fibrils. The BNC-E structure (Figure 2D) appeared partially defibrillated and more porous. However, the fibrils appeared thicker (38–98 nm) compared to BNC-A, and in some regions, individualized nanofibers were clearly visible.^23,40^ As nanocellulose is mainly referred to cellulose that at least one dimension is less than 100 nm, BNC-A and BNC-E could be referred to as nanostructeres.^41,42^

**Figure 2:**
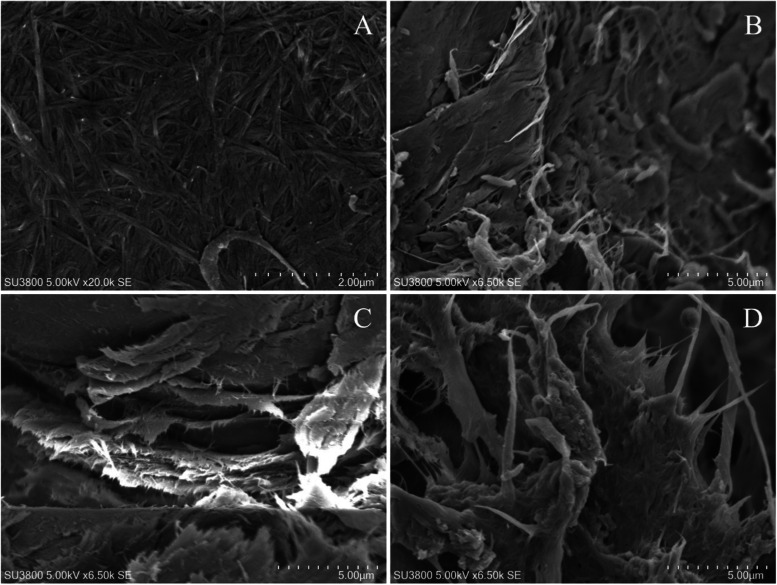
SEM images of (A) BC, (B) MFC, (C) BNC-A, and (D) BNC-E.

Following sugar extraction and BC production, pectin recovery was performed on the SBPR fraction using nitric acid-assisted extraction, yielding 9.8 ± 1.3% pectin. The yield of pectin is directly influenced by extraction parameters such as temperature and time,^11^ with pH and acid type being the most critical factors.^2,43^ The pectin yield of SBP ranges within 6.3 to 33.5%, dependent on several factors of the extraction process.^44,45^ Relevant studies report pectin yields from SBP of up to 12.9% when using nitric acid at lower pH values (pH 1.5).^11^ Higher yields (up to 27.4%) are often achieved using organic acids, such as citric or tartaric acid. However, these strategies may require longer extraction times or elevated temperatures due to the lower dissociation constants of organic acids compared to strong mineral acids like nitric acid, which dissociates completely in solution and can facilitate faster pectin extraction.^46^ Nitric acid is particularly interesting, as it can be produced through novel and eco-friendly processes such as a plasma bubble reactor via an electrolyte-regulation strategy.^47^

In terms of GalA content, the obtained value in this study (76.9 ± 1.4%) falls within the range reported in previous works (61–86%), indicating the high purity of the final material.^11^ Furthermore, the pectin meets and exceeds the EU Regulation No. 231/2012 threshold of 65% GalA for food-grade pectin, suggesting its suitability for food applications.^46,48^ The extracted pectin is classified as high methoxy pectin (HMP) based on its DE (>50%) of 73.2 ± 2.5%, which enables strong gel formation in high-sugar and acidic environments. This property makes it particularly suitable for applications in confectionery and pharmaceutical coatings, where gelling and stabilizing capacities are essential.^11^ This biorefinery approach demonstrates the efficient valorization of SBP, where free sugars are utilized for BC production and the pectin-rich residue serves as a raw material for high-value pectin extraction. This strategy contributes to waste minimization while enabling the production of functional biopolymers for food, pharmaceutical, and biotechnological applications. Pectin derived from SBP, including residual solids utilization have been previously reported in the literature.^2,11^ As the residual biomass after pectin extraction is rich in insoluble fibers, further utilization could be applied to obtain value-added products such as cellulose.^49^ Hassan et al.^49^ investigated the effect of pectin extraction methods from SBP on cellulose nanofibers, showing that acid hydrolysis improved fibrillation and crystallinity, while enzyme hydrolysis yielded nanofibers with better redispersion, mechanical properties, and water resistance.

### Biobased Film Characterization

#### ATR-FTIR

Figure 3A shows the FTIR spectra of SBP pectin, MFC, BNC-A, and BNC-E. All the polysaccharides present a wide band between 3600 and 3000 cm^–1^, which is characteristic of the O–H stretching vibration and hydrogen bonding. The band at 2970–2840 cm^–1^ corresponds to the asymmetric stretching vibrations of CH, CH_2_, and CH_3_ groups present in the polysaccharide molecules.^14^ In the local symmetry region (1500–1200 cm^–1^), primarily observed are the deformational vibrations of groups exhibiting local symmetry. In contrast, the fingerprint region (1200 to 800 cm^–1^) encompasses characteristic absorptions associated with the C–O and C–C bonds.^50^

**Figure 3:**
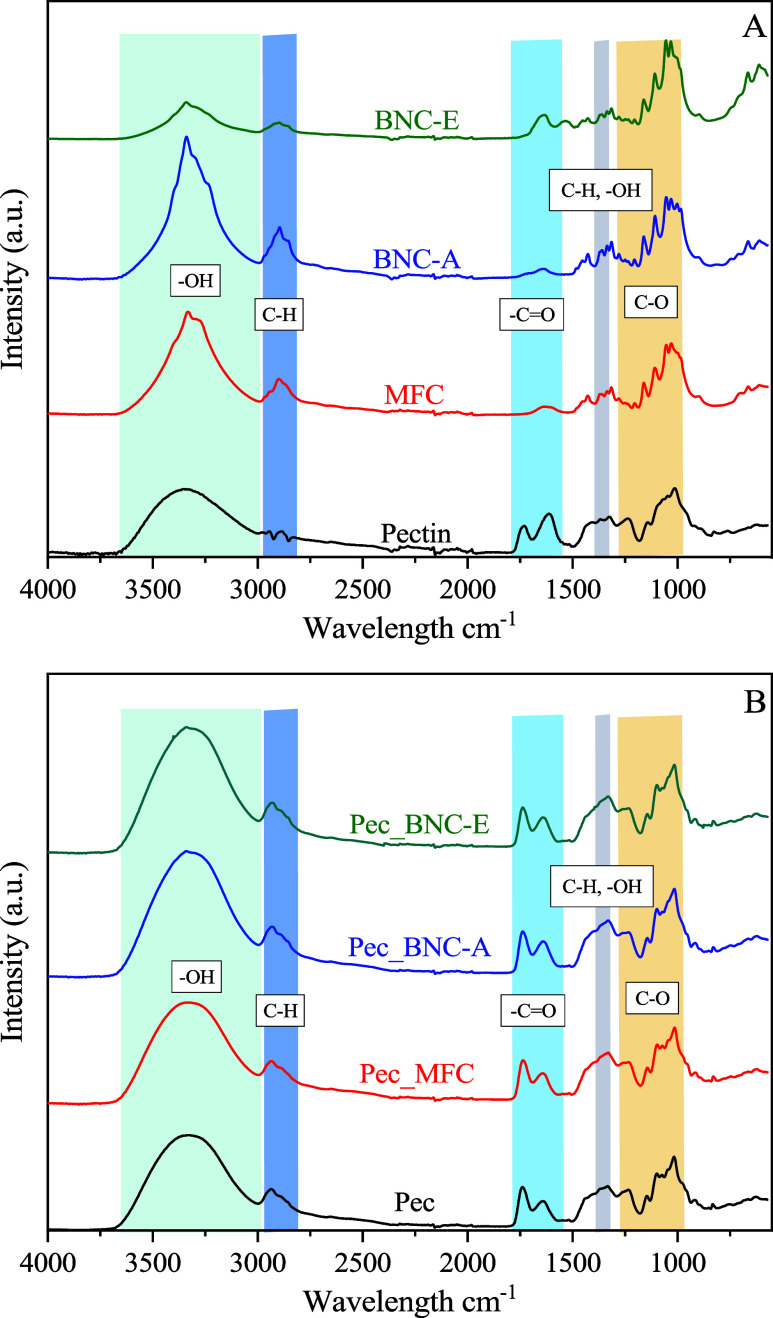
ATR-FTIR spectra of (A) pectin, MFC, BNC-A, and BNC-E, and (B) Pec films reinforced with different cellulose derivatives.

Pectin presents two characteristic peaks at 1730 and 1612 cm^–1^, which are indicative of the C=O group present in the ester (−COOCH_3_) and carboxylate (−COO−) groups.^11^ The peaks of the SBP pectin at around 1367 and 1325 cm^–1^ correspond to the symmetric C–H deformation and C–H wagging, which are coupled with O–H in-plane deformation.^51^ The peak at 1236 cm^–1^ is typically associated with the asymmetric stretching vibrations of C–O–C bonds in the glycosidic linkages of the pectin backbone.^52^ The peak at 1140 cm^–1^ is related to the C–O–C bonds of the glycosidic groups. The peaks at around 1047 and 1013 cm^–1^ are characteristic for asymmetric stretching vibration of the C–O–C bonds and C–O stretching vibrations in polysaccharides, due to the presence of sugars on the GalA backbone.^11^

The DM of the resulting pectin was estimated to be 69.8% (based on the peaks at 1730 and 1612 cm^–1^), being slightly lower than the DE received from the titrimetric method (73.2%). This difference between DM and DE values may be attributed to the presence of proteins in the sample. Protein amide stretching vibrations contribute to absorption bands around 1650 and 1543 cm^–1^, which can interfere with the FTIR-based determination of DM.^53^ Matrix effects, such as the presence of acetyl esters naturally associated with the pectin structure, may undergo saponification during the titration process, leading to an overestimation of the DE. In addition to these matrix-related interferences, inherent methodological limitations contribute to the variability between titration and FTIR results. While titration provides a direct estimation of ester groups based on chemical reactivity, it lacks selectivity in complex samples, as other saponifiable or acidic components may influence DE values. Conversely, FTIR relies on the accurate assignment of characteristic peaks and is sensitive to spectral overlaps and baseline shifts, which can compromise the estimation of DM.^54,55^

The cellulosic samples (MFC, BNC-A and BNC-E), exhibit characteristic peaks at approximately 1427 cm^–1^ (C–H bending vibrations), 1360 cm^–1^ (C–OH in-plane bending vibrations), 1315 cm^–1^ (CH_2_ wagging vibration), and 1054 cm^–1^ (C–O–C skeletal vibrations).^56–58^ The peaks at 1205 and 1160 cm^–1^ correspond to the symmetric and asymmetric stretching vibrations of C–O–C in β-1,4 glycosidic linkages, respectively.^59,60^ Additionally, the peaks observed at ∼1108 and 1031 cm^–1^ are attributed to ring asymmetric valence vibrations and C–O stretching vibrations, respectively.^57^ Characteristic peaks at 1003 and 984 cm^–1^ are presented on the BNC-A sample, attributed to the structural modifications caused by the removal of amorphous regions. The solder around 810 cm^–1^ could be attributed to the S–O stretching vibrations, due to the sulfation of hydroxyl groups during the sulfuric acid hydrolysis.^21^ The removal of amorphous regions, thus the crystallinity of cellulose, is associated with the peak ratio at ∼1430 and 898 cm^–1^, which is higher in BNC-A rather than BNC-E.^57,61^ Modification of BC through sulfuric acid hydrolysis selectively removes amorphous domains, resulting in higher crystallinity values (>92%).^21^ In contrast, enzymatic hydrolysis disrupts the crystalline regions of BC, leading to a lower crystallinity index (65–70%) compared to acid-hydrolyzed BC.^62^

The potential interactions between pectin and cellulosic samples (MFC, BNC-A, and BNC-E) were investigated (Figure 3B). The characteristic peaks of pectin molecules are presented in all of the biobased films. The absorption peak of −OH vibration on Pec_BNC-A and Pec_BNC-E was shifted to a higher wavenumber (from 3332 to 3340 cm^–1^), indicating hydrogen bond distribution. The peak of −C=O stretching was shifted from 1647 to 1640 cm^–1^ with the addition of BNCs, indicating interactions between C=O groups of pectin and BNC-A. Electrostatic repulsion between pectin and BNCs is probably formulated, as both components carry a negative charge.^63^ No significant interactions were observed in the FTIR spectra between SBP pectin and MFC. The interactions between pectin and cellulose are complex and influenced by the source of pectin. In the case of SBP pectin, arabinan side chains are abundant and may influence gelation, or hydrogen bonding capabilities, playing a crucial role in affecting interactions with cellulose and thus performance of composite films.^6^

#### Optical Properties

The color parameters L*, a*, and b*, total color difference, whiteness index, and yellowness index of the produced films are presented in Table 1. All films exhibited similar L* values (*p > 0.05*), indicating that the addition of MFC and BNCs did not significantly alter the film lightness. The observed L* values align with those reported in previous studies for various Pec films.^64,65^ The a* and b* coordinates remained constant across all formulations (*p > 0.05*), confirming that the addition of cellulose-based reinforcements did not induce a noticeable color shift. Among the tested films, Pec_BNC-A exhibited the highest b* value (14.7), indicating increased yellowness compared to both Pec (control) and Pec_MFC films. This trend was further supported by the yellowness index, which was higher in Pec_BNC-A (411, *p > 0.05*) compared to the Pec films (231). The whiteness index decreased in Pec_BNC-A (*p =* 0.22), also reflecting a shift toward a more yellowish appearance. This change in optical properties may be attributed to partial sulfation occurring during the hydrolysis of BC with H_2_SO_4_. Sulfation has been previously reported to induce structural modifications in cellulose, altering its light absorption properties and enhancing yellow pigmentation due to the introduction of sulfate ester groups.^21^ These chemical modifications may also influence intermolecular interactions between pectin and cellulose, potentially affecting the film’s optical homogeneity and transparency.

**Table 1:** Color and UV–vis Properties of Pec Films Reinforced with MFC and BNCs^a^

parameters	Pec	Pec_MFC	Pec_BNC-A	Pec_BNC-E
L*	81.8 ± 1.8^a^	81.4 ± 2.8^a^	81.0 ± 2.0^a^	81.8 ± 1.8^a^
a*	3.4 ± 0.5^a^	3.7 ± 0.8^a^	3.7 ± 0.6^a^	3.5 ± 0.5^a^
b*	11.1 ± 2.7^a^	12.7 ± 4.9^a^	14.7 ± 4.0^a^	13.3 ± 4.0^a^
Δ*E*		4.6 ± 3.9^a^	4.7 ± 3.4^a^	4.1 ± 2.9^a^
WI	78.4 ± 2.9^a^	77.1 ± 5.1^a^	75.6 ± 4.0^a^	77.1 ± 3.8^a^
YI	231.0 ± 104^a^	331.0 ± 263^a^	411.0 ± 210^a^	339.0 ± 188^a^
T%	23.6 ± 1.7^b^	23.0 ± 2.7^ab^	18.7 ± 2.2^a^	20.1 ± 1.7^ab^
UV_A_-blocking %	92.0 ± 1.8^a^	91.8 ± 0.9^a^	93.9 ± 2.2^ab^	96.4 ± 0.1^b^
UV_B_-blocking %	97.6 ± 0.2^a^	97.8 ± 0.1^ab^	98.0 ± 0.1^b^	100.0 ± 0.0^c^

a Statistically significant differences (*p* < 0.05) for the same parameter within the same row are indicated with different letters (a–c). L*, lightness; a*, redness; b*, yellowness; Δ*E*, total color difference; WI, whiteness index; YI, yellowness index; *T*, transparency.

#### Transparency and UV–vis Transmittance

Transparency (*T*%) is a key characteristic in food packaging, as it determines the visual appeal and product visibility.^66,67^ Light transmittance directly influences material transparency and its ability to shield packaged food from UV radiation.^68^ Conventional petroleum-based packaging materials, such as low-density polyethylene (LDPE) and polypropylene (PP), exhibit high optical transparency but lack effective UV-blocking properties, thereby offering limited protection against the oxidative deterioration of photosensitive foods.^66,67^ Packaging materials with more than 90% transmittance are characterized as highly transparent, whereas 80% transmittance is an accepted threshold for transparent packaging materials.^67^

Representative images of the formulated films are presented in Figure 4A, demonstrating their transparency. Transparency was significantly affected by cellulose-based reinforcing. However, all of the transmittance values at 600 nm (the midpoint of the visible range) were <80%, indicating the formation of semitransparent materials (Figure 4B).^67^ The light transmission spectra (200–800 nm) of the studied films (Figure 4B) revealed that Pec films exhibited the highest transparency (23.6%), while incorporating BNCs resulted in decreased values (18.7–20.1%) (Table 1). Notably, Pec_BNC-A films showed the lowest transparency (*p < 0.05*), which is consistent with the observed increase in the yellowness. This reduction can be attributed to the high biocompatibility of BNCs with pectin, leading to fewer aggregates and improved polymer–polymer interactions. This enhanced molecular integration increases light-scattering effects, thereby lowering overall transparency.^20,60,69^ In contrast, MFC-containing films maintained similar transparency levels to Pec films (*p > 0.05*), likely due to their long fibrillar network, which promotes a more open and less light-scattering structure.^70,71^ These findings are consistent with previous studies that have demonstrated that cellulose-based reinforcements typically reduce transparency in pectin films due to light-scattering and aggregation phenomena. The high compatibility of BNCs with the polymer matrix can further intensify this effect by modifying the optical path of transmitted light.^72–74^ Overall, films presented transparency values (18.7–23.6%), consistent with previous relevant studies.^14,75^

**Figure 4:**
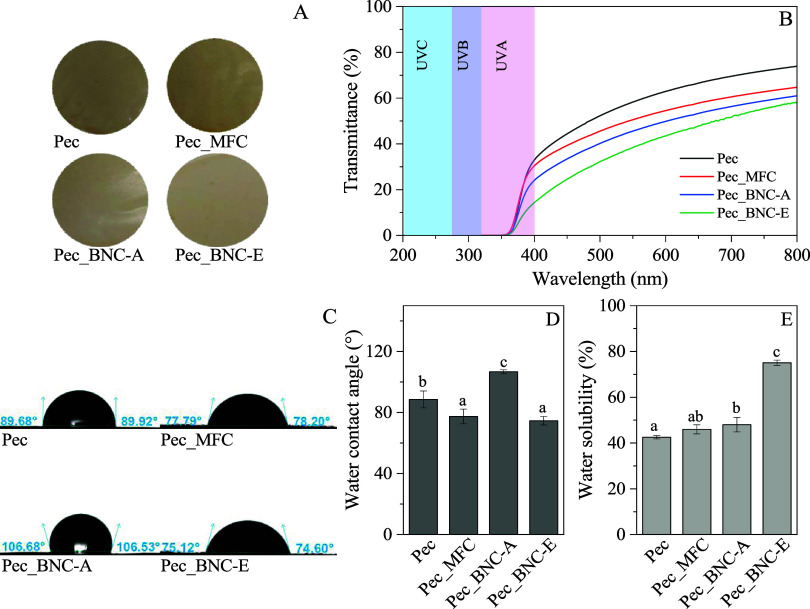
Properties of pectin films reinforced with MFC, BNC-A, and BNC-E. (A) Representative images of the films, (B) UV–vis transmittance spectra, (C) images of contact angles, (D) contact angle values, and (E) solubility values. Statistically significant differences (*p < 0.05*) are indicated using different subscript letters (a–c) within the same figure (D and E).

The UV transmittance properties of the films are presented in Table 1. All films exhibited low transmittance in the UVA/UVB range (280–400 nm), while no transmittance was detected in the UVC range (200–280 nm). Transmittance values of films (44–63%) are consistent with previously reported values for pectin-based films, which typically demonstrate strong UV-blocking capacity due to their inherent composition.^14,75^ Pec films displayed very good UV-blocking properties (92% for UVA and 97.6% for UVB radiation). This can be attributed to the presence of ferulic acid in SBP, which acts as a natural UV-absorbing compound.^16^ SBP-derived pectin is particularly rich in feruloyl groups, existing in both free ferulic acid and esterified forms, which are covalently linked to pectin side chains. These structures enhance the material’s antioxidant activity and UV absorption capacity, contributing to its strong shielding effect against UV radiation.^17,76^

The incorporation of BNCs further enhanced the UV-blocking capacity of the films (Table 1), likely due to additional BNC-induced scattering effects. MFC-containing films did not significantly alter UV transmittance compared with neat Pec films (*p > 0.05*). This suggests that the structural differences between MFC and BNCs play a key role in UV absorption efficiency. The transparency and UV-blocking properties of films are affected by several factors, including crystallinity, surface properties, and the density of the polymer chain structures.^33,66^ The UV-shielding efficiency of cellulose-reinforced films aligns with previous studies on biobased films cast with gelatin as the matrix and supplemented with cellulose nanocrystals derived from eucalyptus kraft pulp^77^ and soy protein formulations reinforced with cellulose from licorice residue.^72^ The above-mentioned results indicate the strong UV-barrier efficiency of the biobased films, suggesting their potential as promising candidates for active food packaging applications. Effective UV shielding can delay degradation by preventing oxidative damage, color changes, nutrient loss, and microbial spoilage, ultimately extending the shelf life of packaged food products.^78^ This characteristic in accordance with the principles of active packaging, which encompasses protection against environmental stressors that degrade food quality during storage.^79^

#### Water Barrier Properties and Hydrophilicity

The hydrophobicity of the films was evaluated by contact angle measurements, while the solubility and WVP values were assessed to determine their suitability for food packaging applications (Figure 4). Neat Pec films exhibited a contact angle of 88.6° (<90°), indicating slightly hydrophilic behavior. Pec films are inherently hydrophilic due to the abundance of hydroxyl and carboxyl functional groups, which readily interact with water molecules via hydrogen bonding.^15^ However, HMP used in film formulation introduces a higher DE (>50%), where carboxyl groups are methyl-esterified, limiting hydrogen bonding sites and enhancing hydrophobicity.^80^

The incorporation of MFC and BNC-E into the films further enhanced the surface hydrophilicity, as indicated by a reduction in contact angle values. This effect is likely due to the presence of hydroxyl groups from MFC and BNC, which can form hydrogen bonds with water, thereby increasing water affinity.^14,25^ Notably, BNC-A-reinforced films exhibited a significantly higher contact angle of 106.7° (>90°), rendering them hydrophobic. The impact of BC treatment on hydrophobicity was evident. BNC-E was obtained via enzymatic hydrolysis, which partially degrades the amorphous cellulose regions while preserving the crystalline domains. This treatment enhances fiber dispersibility and exposes additional hydroxyl groups, thereby increasing the material’s affinity for water molecules.^81–83^ Consequently, Pec_BNC-E films exhibited the lowest contact angle (74.6°). In contrast, BNC-A was obtained via H_2_SO_4_ hydrolysis, which introduces sulfate ester (–OSO_3_^–^) groups onto the cellulose surface. These sulfated functional groups increase electrostatic repulsion between cellulose chains, reducing water accessibility and promoting hydrophobic behavior.^84^ Higher crystallinity values reduce the number of available hydroxyl sites for water interaction, making BNC-A less prone to water absorption and BNC-E more water-soluble.^85^

The surface wettability of Pec-based films reinforced with MFC and BNC derivatives aligns well with trends reported in the literature for conventional and biodegradable packaging films. While traditional fossil-based polymers such as polypropylene and polyethylene generally exhibit hydrophobic surfaces with contact angles around 90–100°, biodegradable polylactic acid (PLA)-based films typically show lower contact angles (73 to 81°).^86^

The addition of MFC and BNC-A led to a moderate increase in solubility, rising from 42.5% (Pec films) to 46.0% (Pec_MFC films) and 48.0% (Pec_BNC-A films). In contrast, BNC-E incorporation resulted in a notable increase in the film’s solubility (75.1%), confirming its hydrophilic nature. This aligns with the increased availability of hydroxyl groups due to enzymatic hydrolysis, which enhances the water interaction and swelling capacity. WVP values were slightly higher in cellulose-reinforced films (1.83 × 10^–7^ – 2.07 × 10^–7^ g/m·h·Pa) compared to neat Pec films (1.78 × 10^–7^ g/m·h·Pa), though the differences were not statistically significant (*p > 0.05*). This indicates that while cellulose-based reinforcements influenced water interactions on the film surface, they did not substantially alter the overall moisture permeability of the films. The observed solubility and WVP values are consistent with previously reported studies on Pec films.^14,87^ The different trends observed between hydrophobicity and WVP in the Pec_BNC-A films can be attributed to the distinct mechanisms governing these two properties. Contact angle measurements are related to the surface physicochemical properties, whereas WVP reflects moisture transport through the entire film matrix and is influenced by the film’s internal structure, such as porosity, and molecular organization, which governs water vapor diffusion through the material.^88,89^ Thus, although BNC-A incorporation altered the surface polarity, it may not have significantly modified the bulk microstructure or reduced continuous pathways for water vapor transmission. This suggests that the internal core of the film matrix was not substantially affected by BNC-A incorporation, leading to minimal changes in overall moisture permeability despite the more hydrophobic surface. Understanding both surface chemistry and internal architecture remains essential for designing films with optimized moisture barrier properties.

The ability to tailor water barrier properties of biobased films through cellulose modification provides valuable insights for food packaging design, addressing different food preservation needs. Hydrophilic films (Pec_BNC-E, Pec_MFC) may be beneficial for applications where the release of bioactive compounds that are water-activated is needed, while hydrophobic films (Pec_BNC-A) could serve as effective moisture barriers, making them ideal for extending shelf life and protecting fat-rich food products from water-induced spoilage. However, achieving optimal moisture resistance in Pec films remains a key challenge due to the inherently hydrophilic nature of water-soluble polysaccharides.

Hydrophobicity and wettability are critical parameters influencing the water barrier effectiveness of biobased packaging films.^90^ To enhance these properties, pectin-cellulose composites with various additives have been explored. Specifically, pectin is blended with nanostructured or modified BC, to achieve desired structural and chemical characteristics of the cellulose used.^19^ Yang et al.^91^ demonstrated that reinforcing pectin-based films with carboxylated cellulose nanocrystals-stabilized oregano essential oil emulsions enhanced mechanical and hydrophobic properties. Likewise, Viana et al.^19^ modified BC into nanofibrils through TEMPO-oxidation, which, when integrated into pectin-rich films, enhanced moisture resistance and overall film integrity. Efthymiou et al.^20^ synthesized BC nanocrystals via H_2_SO_4_-assisted hydrolysis, which served as reinforcing agents in protein films, significantly reducing solubility and WVP. Pectin films with curcumin and sulfur nanoparticles exhibited increased hydrophobicity and improved UV-blocking properties.^87^ Pectin-cellulose films enriched with sodium borate were investigated as coatings on conventional packaging materials, demonstrating enhanced humidity-regulating properties.^12^ Additionally, incorporating dialdehyde or cotton-derived cellulose nanocrystals into pectin-based films enhanced their mechanical strength, UV resistance, water barrier properties, and enabled controlled oil release.^13,92^ The final output was biobased films with enhanced water barrier/mechanical properties, with controlled oil release.

#### Mechanical Properties

The mechanical properties of the films are summarized in Figure 5. The tensile properties of Pec films included tensile strength at 9.1 MPa, elongation at break of 14.6%, and Young’s modulus of 169 MPa, consistent with values reported in previous studies.^91,93,94^ Upon reinforcement of the films with MFC, no significant change in tensile strength was observed (8.0 MPa). However, a decrease in elongation at break (9.3%) and a notable increase in Young’s modulus (263 MPa) were recorded. These results indicate a transition to a film of lower ductility, which aligns with findings from earlier studies indicating that poor dispersion of MFC can lead to mechanical property changes in composite films.^70^ This pattern of increased stiffness and reduced ductility reflects the challenges typically encountered when incorporating reinforcing agents into biobased matrices, especially when dispersion is suboptimal. Incorporating BNC-A into the pectin matrix significantly improved the mechanical profile (*p < 0.05*), with values of tensile strength (12.7 MPa), elongation at break (22.5%), and Young’s modulus (260.7 MPa), being consistent with previous relevant studies.^14,20^ These improvements can be attributed to the rigid network provided by the sulfated nanocrystals in BNC-A, which create strong interfacial interactions/compatibility with the pectin matrix, resulting in better dispersion and distribution of the BNCs throughout the films. This structure enhances both strength and ductility.^20^ In contrast, BNC-E incorporation exhibited a significant reduction in tensile strength (5.4 MPa), whereas no significant differences were observed on elongation at break and Young’s modulus values (*p > 0.05*) compared to Pec films. BNC-A, produced via H_2_SO_4_ hydrolysis, typically exhibits a crystallinity higher than that of BNC-E, which likely contributes to its higher tensile strength and resistance to deformation. The crystalline regions in BNC-A form a structured network within the pectin matrix, improving the film’s mechanical integrity. On the other hand, the lower crystallinity and more amorphous structure of BNC-E, resulting from enzymatic treatment, result in weaker interfacial interactions with the pectin matrix, reducing the overall strength of the film under stress. The less ordered arrangement of BNC-E fibrils contributes to the lower elongation at break observed in comparison to BNC-A films, indicating reduced ductility.^59,62^

**Figure 5:**
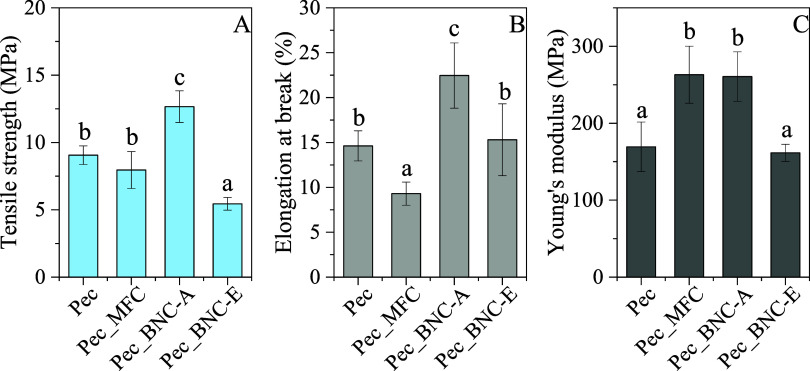
Mechanical properties of the Pec films. (A) tensile strength, (B) elongation at break, (C) Young’s modulus. Statistically significant differences (*p < 0.05*) are indicated using different letters (a–c) within the same figure.

The selection of the most suitable film for food packaging applications depends on multiple factors, including mechanical performance, barrier properties, sustainability profiles, and specific product protection requirements. In this context, Pec/BNC-A films demonstrate a distinctive and promising mechanical profile. With a tensile strength of 12.7 MPa, elongation at break of 22.5%, and a Young’s modulus of approximately 261 MPa, these films offer a well-balanced combination of stiffness and flexibility, making them suitable for targeted applications in biodegradable and active packaging. When compared with conventional fossil-based plastics, Pec/BNC-A films show a competitive performance. Their tensile strength lies within the range of LDPE (8–31 MPa), and their modulus is comparable to the lower end of LDPE (200–500 MPa), indicating sufficient structural integrity and flexibility for many packaging uses. Although their tensile strength is lower than that of PP (31–43 MPa) and especially PET (220–270 MPa), this reflects the expected trade-offs when transitioning toward biobased, environmentally conscious materials. Compared to biodegradable synthetic polymers, Pec/BNC-A films stand out for their notably higher elongation at break than PLA (4–7%) and PHB (1–15%), reflecting greater ductility, a desirable feature in flexible packaging formats. While their modulus (∼261 MPa) is significantly lower than that of PLA (∼1280 MPa) and PHB (∼1200 MPa), it is considerably higher than that of PBS (48 MPa), suggesting a favorable stiffness–flexibility balance. Although the tensile strength of Pec/BNC-A films remains below that of PLA (44–59 MPa) and PBS (34 MPa), these materials offer high tunability through formulation and processing techniques.^95^

Importantly, the biobased nature and renewable origin of Pec/BNC-A films align with sustainability goals in the packaging industry. Their compatibility with emerging nonthermal processing technologies, such as high-pressure processing, cold plasma, microwave, and ultrasound treatments, further enhances their potential. These technologies can be employed to improve both mechanical and barrier properties, making Pec/BNC-A films a versatile and forward-looking solution for modern, sustainable food packaging systems.

The present strategy on upcycling of agro-industrial byproducts underscores their potential in sustainable biopolymer production for innovative solutions in high-demanding sectors. The reinforcement of SBP pectin with cellulose derivatives (BNCs or MFC) led to the development of biobased, sustainable film formulations with tailored water and light barrier properties and mechanical profile. The films exhibited satisfying transparency, crucial for visual monitoring of packaged food, while maintaining esthetic appeal. The strong UV-blocking properties of biobased films, and particularly of BNCs-reinforced ones, provide food quality preservation by mitigating UV-induced degradation. The hydrophilicity of the composite films was effectively tailored by selecting the appropriate cellulose type as a reinforcing agent, providing a strategy for customizing material properties for specific applications. BNC-A films, which exhibited the highest contact angle (106.7°), could be suitable for moisture-resistant food packaging, providing extended shelf life for the products (e.g., bakery, cereals). The mechanical properties of the films were notably improved with the addition of BNC-A, probably attributed to the strong interfacial interactions and compatibility between BNC-A and the pectin matrix. Water vapor permeability remained similar across all films, indicating that the cellulose reinforcements did not significantly alter the moisture permeability, which is essential for packaging applications requiring precise moisture control. Overall, this study demonstrated the versatility of Pec films reinforced with cellulose derivatives to develop satisfactory eco-friendly food packaging. Particularly, BNC-A-reinforced pectin films present a viable way to partially substitute traditional petroleum-based packaging, assisting in reducing the overall use of synthetic materials and supporting the transition toward a more circular and sustainable food industry.
